# Comparative Analysis of the Main Bioactive Components of San-ao Decoction and Its Series of Formulations

**DOI:** 10.3390/molecules171112925

**Published:** 2012-11-01

**Authors:** Xiaoyun Shu, Yuping Tang, Chenxue Jiang, Erxing Shang, Xinshen Fan, Anwei Ding

**Affiliations:** Jiangsu Key Laboratory for High Technology of TCM Formulae Research, Nanjing University of Chinese Medicine, Nanjing 210046, China

**Keywords:** San-ao decoction, formulations, Ephedrae Herba, Armeniacae Semen Amarum, Glycyrrhizae Radix et Rhizoma, HPLC-DAD

## Abstract

A high performance liquid chromatographic (HPLC) method with diode array detection (DAD) was established for simultaneous determination of seven main bioactive components in San-ao decoction and its series of formulae (San-ao decoction, Wu-ao decoction, Qi-ao decoction and Jia-wei San-ao decoction). Seven compounds were analyzed simultaneously with a XTerra C_18_ column (4.6 mm × 250 mm, 5 µm) using a linear gradient elution of a mobile phase containing acetonitrile (A) and a buffer solution (0.02 mol/L potassium dihydrogen phosphate and adjusted to pH 3 using phosphoric acid) (B); the flow rate was 1.0 mL/min. The sample was detected with DAD at 210, 254 and 360 nm and the column was maintained at 30 °C. All the compounds showed good linearity (r^2^ > 0.9984) in the tested concentration range. The precisions were evaluated by intra-day and inter-day tests, and relative standard deviation (R.S.D.) values within the range of 0.83%–2.53% and 0.64%–2.77% were reported, respectively. The recoveries of the quantified compounds were observed to cover a range from 95.34% and 104.82% with R.S.D. values less than 2.72%. The validated method was successfully applied for the simultaneous determination of seven main bioactive components including ephedrine (**1**), amygdalin (**2**), liquiritin (**3**), benzoic acid (**4**), isoliquiritin (**5**), formononetin (**6**) and glycyrrhizic acid (**7**) in San-ao decoction and its series of formulae. The results also showed a wide variation in the content of the identified active compounds in these samples, which could also be helpful to illustrate the drug interactions after some herbs combined in different formulations.

## 1. Introduction

The composition of Traditional Chinese Medicines (TCMs) is at odds with modern medicine. TCM uses formulae that contain several herbs thought to act in unison to restore what TCM practitioners call the patient’s “balance”, and some series of formulae (named analogous formulae), groups of formulae with some similarity in medical compositions (combinations) and indications (syndromes), are usually used in TCM clinical practice. Study on series of formulae is an efficient approach to understanding the scientific basis of formulae assembly and syndrome differentiation in TCM, which can also be helpful for elucidating the complex series of Chinese medicine prescriptions and developing new medicines [1,2,3,4,5,6].

San-ao decoction (SAD) is a representative TCM formula for asthma, which is comprised of three herbs, including Ephedrae Herba (EH), Armeniacae Semen Amarum (AS) and Glycyrrhizae Radix et Rhizoma (GR) at the ratio of 4:10:4. In ancient times, neither the root and eustipes of EH, nor the seed coat of AS were removed, and GR did not need to be processed with honey, thus the formula is not too transpirative or astringent, and was named as “San-ao” decoction. A series of formulae were employed for treatment of asthma diseases in China, of which 42% were based on SAD, such as Wu-ao decoction (WAD), Qi-ao decoction (QAD) and Jiawei San-ao decoction (JSD) [7]. All of these formulae with their constituent herbs are summarized in Table 1. Nowadays, SAD and its series of formulae are widely used in clinical practice for treating bronchitis caused by bacterial and viral infections. Histopathologic examination showed that SAD suppressed the neutrophil infiltration into lung tissue, which indicated that the antiasthmatic effect of SAD is contributed by its bronchodilator effect and inhibition of neutrophils in the airway [8]. SAD may have a inhibitory effect on airway inflammation of asthmatic rats by regulating Th1/Th2 transfer factors [9]. Our recent research showed that SAD and its series of formulae could alleviate normal human bronchial epithelial cell damage and suppress airway inflammation by various ways [10,11,12,13,14,15,16,17,18].

**Table 1:** The composition of San-ao Decoction and its series of formulae.

Formulae Name	Composition
SAD	Ephedrae Herba (EH, 4 g), Armeniacae Semen Amarum (AS, 10 g), Glycyrrhizae Radix et Rhizoma (GR, 4 g)
WAD	Ephedrae Herba (EH, 4 g), Armeniacae Semen Amarum (AS, 10 g), Glycyrrhizae Radix et Rhizoma (GR, 4 g), Schizonepetae Herba (SH, 8 g),Platycodonis Radix (PR, 8 g)
QAD	Ephedrae Herba (EH, 4 g), Armeniacae Semen Amarum (AS, 10 g), Glycyrrhizae Radix et Rhizoma (GR, 4 g), Pinelliae Rhizoma (PR, 10 g),Schisandrae Chinensis Fructus (SC, 6 g), Gypsum Fibrosum (GF, 20 g),Camelliae sinensis Folium (CS, 10 g)
JSD	Ephedrae Herba (EH, 4 g), Armeniacae Semen Amarum (AS, 10 g), Glycyrrhizae Radix et Rhizoma (GR, 4 g), Asari Radix et Rhizoma (AR, 10 g)

Ephedrine, which is the bio-marker constituent in EH, can relax bronchial smooth muscle [19]. Amygdalin had a sedative effect on the respiratory center [20], and benzoic acid could improve the anticancer effects [21]. Glycyrrhizic acid could suppresses Cox-2-mediated anti-inflammatory responses [22]; liquiritin had antidepressant effects [23]; isoliquiritin had antigenotoxic effects [24]; formononetin could reduce production of proinflammatry mediators in LPS-stimulated macrophages, fibroblasts, and intestinal epithelial cells [14]. It was reported that these constituents were the main active compounds of SAD [13,14]. To date, some preliminary research concerning the quantitative analysis of active components in SAD using HPLC methods have been reported [25,26,27,28,29,30]. However, there is little information available in the literature about the variation of the main bio-active constituents in San-ao decoction and its series of formulae, therefore, it is necessary to establish a method for comparative characterization of these active components in these formulae, which is also useful to illustrate the changes of the contents of these marker compounds after herbs are combined into different formulae.

**Figure 1:**
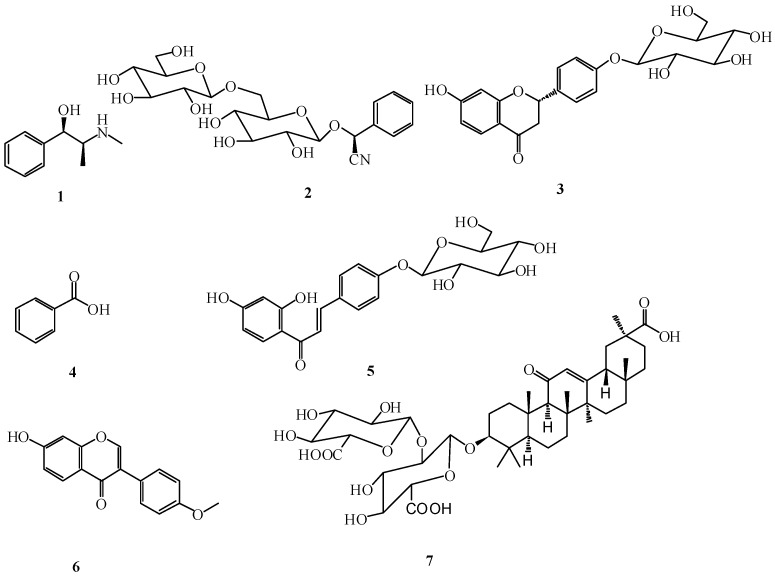
The structures of the reference compounds: ephedrine (**1**), amygdalin (**2**), liquiritin (**3**), benzoic acid (**4**), isoliquiritin (**5**), formononetin (**6**) and glycyrrhizic acid (**7**).

In recent years, some researchers have started to become interested in the compatibility rules of the TCM formulae by comparatively analyzing some of the main bioactive components of different herbs or formulae [31,32,33,34,35,36,37]. This study aimed to develop a direct and convenient high performance liquid chromatographic coupled with diode array detector (HPLC-DAD) method to simultaneously analyze quantitatively the seven main bio-active components in San-ao decoction and its series of formulae, including ephedrine (**1**), amygdalin (**2**), liquiritin (**3**), benzoic acid (**4**), isoliquiritin (**5**), formononetin (**6**) and glycyrrhizic acid (**7**) (Figure 1). The study will lay a foundation to establish the quality standards for these formulae and the results were also helpful to illustrate the drug interactions after some herbs are combined into different formulae.

## 2. Results and Discussion

### 2.1. Method Validation

The chromatographic methods were validated to determine the linearity, LOD, LOQ, intraday and interday precisions, and accuracy. A stock solution containing the seven reference compounds was prepared and diluted to seven appropriate concentrations to obtain the calibration curves, which were plotted after linear regression of the peak areas compared with the corresponding concentrations. The lowest concentration of working solution was diluted with methanol to a series of appropriate concentrations, and aliquots of these diluted solutions were injected into the HPLC for analysis. The LOD was determined at a signal-to-noise (S/N) ratio of 3, and LOQ was determined at a S/N ratio of 10. The calibration curve results indicated that all seven reference compounds showed good linearity (*r*^2^ > 0.9984) within relatively wide concentration ranges. The LODs and LOQs of the seven analytes were 0.10–3.70 and 0.60–11.10 ng, respectively. The linearity, LOD and LOQ values of each analyte are presented in Table 2.

**Table 2:** Regression equation, correlation coefficients, linearity ranges, limits of detection (LOD) and limits of quantification (LOQ) of investigated compounds.

Analytes	Regression equation	*r* ^2^	Linear range (μg)	LOD ^b^ (ng)	LOQ ^c^ (ng)
Ephedrine	^a^ y = 600818x + 78730	0.9997	0.32~20.56	1.60	4.80
Amygdalin	y = 469675x − 4930	0.9986	0.74~47.60	3.70	11.10
Liquiritin	y = 385254x + 5938	0.9984	0.16~10.40	0.80	2.40
Benzoic acid	y = 5493640x − 27349	0.9998	0.02~1.12	0.10	0.30
Isoliquiritin	y = 6553375x − 227224	0.9994	0.10~6.16	0.50	1.50
Formononetin	y = 888962x + 22087	0.9984	0.04~2.56	0.20	0.60
Glycyrrhizic acid	y = 803987x − 134817	0.9987	0.43~27.44	2.15	6.45

^a^ y is the peak area in UV chromatograms monitored at the absorption maximum for each reference compound, x is the compound amount injected (ug). ^b^ LOD refers to the limits of detection. ^c^ LOQ refers to the limits of quantification.

The intraday and interday precisions were investigated by analyzing known concentrations of analytes in six replicates during a single day and by duplicating the experiments on three successive days, respectively. The relative standard deviation (RSD) was taken as a measure of precision. The results indicated that the intra- and interday RSD values of the seven compounds were all lower than 3.0% (Table 3). The repeatability was assessed by analyzing six independently prepared samples using the same method. Sample stability was evaluated at room temperature and analyzed at 0, 2, 4, 8, 12 and 24 h within one day, respectively. The RSD was taken as a measure of repeatability and stability and the results of the seven compounds in SAD are showed in Table 3. Recovery was calculated by spiking accurate amounts of the seven standards at low (80% of the known amounts), medium (same as the known amounts), and high (120% of the known amounts) to SAD sample. The resultant samples were then extracted and analyzed by using the proposed procedure. As shown in Table 3, the recoveries of the seven compounds were in the range of 95.34%–104.82%, which indicated that the methods were accurate enough for the determination of these compounds in SAD and its series of formulae.

**Table 3:** Precision, repeatability stability and recovery of the seven analytes.

Analytes	Precision (R.S.D., %)		Repetability **	Stability **	Recovery (%, *n* = 9)	
	Intra-day	Inter-day	(R.S.D., %,	(R.S.D., %,	Mean	R.S.D. (%)
	( *n* = 6)	( *n* = 3)	*n* = 6)	*n* = 6)		
Ephedrine	1.21	1.69	0.56	1.82	103.13	2.26
Amygdalin	1.84	2.77	1.72	2.13	104.82	2.72
Liquiritin	0.93	0.64	1.22	1.85	96.27	1.55
Benzoic acid	2.53	2.19	1.84	1.32	97.14	2.69
Isoliquiritin	1.79	1.94	1.84	2.04	96.26	1.41
Formononetin	1.22	1.73	0.95	2.17	95.34	2.38
Glycyrrhizic acid	0.83	0.69	0.91	2.59	98.39	1.16

### 2.2. Sample Analysis

**Figure 2:**
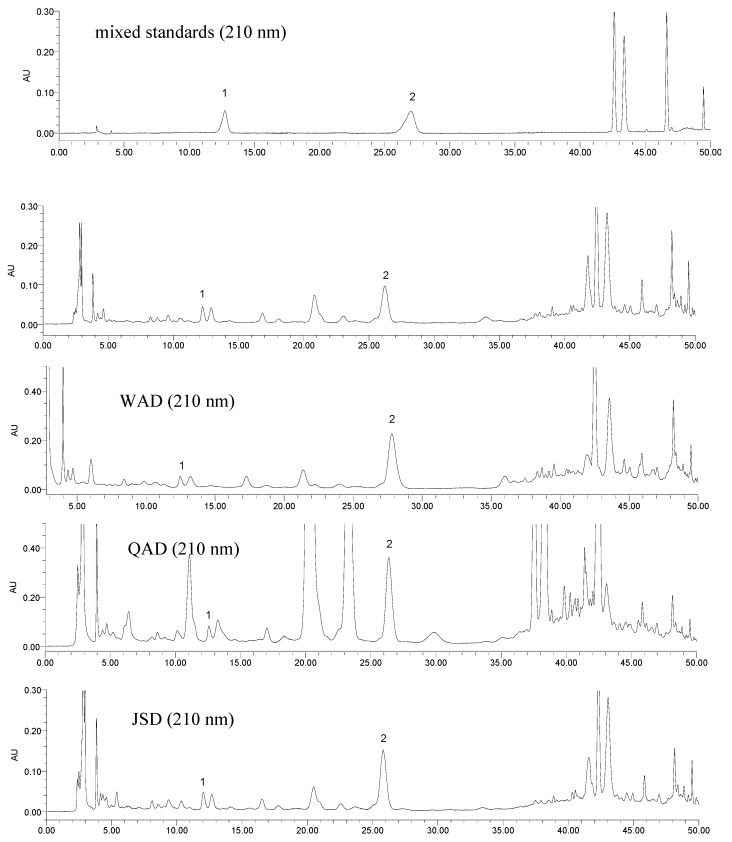
Typical HPLC chromatograms of mixed standards and the investigated samples at 210 nm. ephedrine (**1**), amygdalin (**2**), liquiritin (**3**), benzoic acid (**4**), isoliquiritin (**5**), formononetin (**6**) and glycyrrhizic acid (**7**).

**Figure 3:**
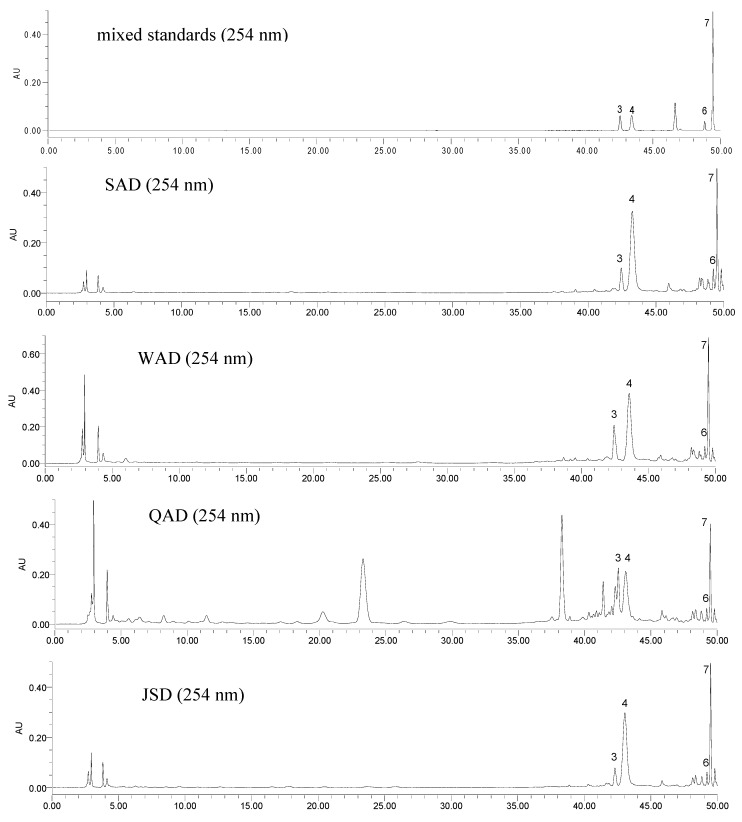
Typical HPLC chromatograms of mixed standards and the investigated samples at 254 nm. ephedrine (**1**), amygdalin (**2**), liquiritin (**3**), benzoic acid (**4**), isoliquiritin (**5**), formononetin (**6**) and glycyrrhizic acid (**7**).

**Figure 4:**
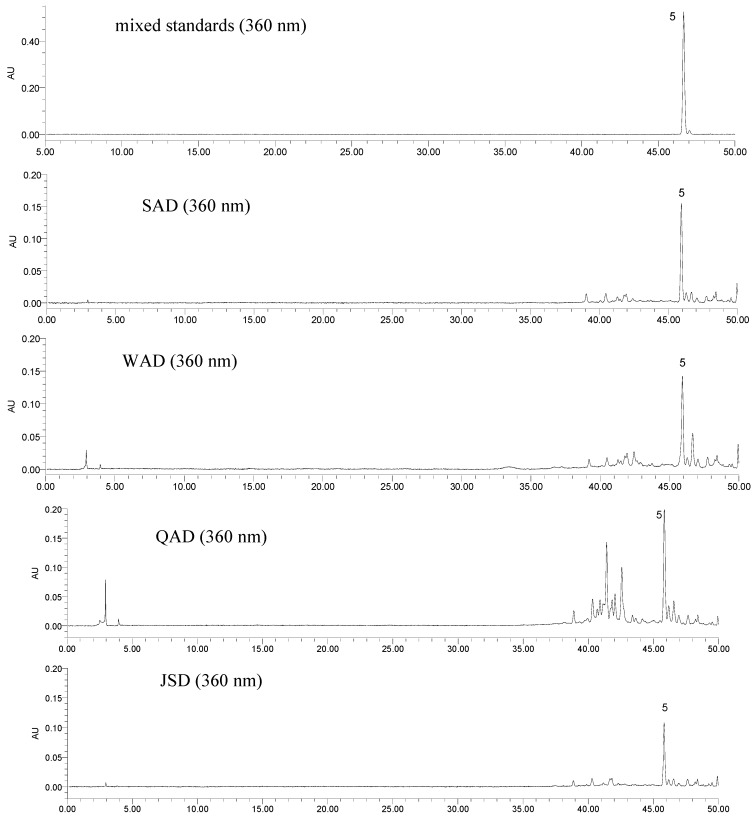
Typical HPLC chromatograms of mixed standards and the investigated samples at 360 nm. ephedrine (**1**), amygdalin (**2**), liquiritin (**3**), benzoic acid (**4**), isoliquiritin (**5**), formononetin (**6**) and glycyrrhizic acid (**7**).

The established analytical method was then subsequently applied to a simultaneous determination of the seven markers of SAD and its series of formulae. Typical HPLC chromatograms of mixed standards and the investigated samples at 210, 254 and 360 nm are shown in Figure 2, Figure 3 and Figure 4, respectively. The results (Table 4) showed that the content of the seven constituents displayed striking differences among the analyzed samples. The contents variation of compounds **1**–**7** were as follows: for compound **1**: QAD > JSD > SAD > WAD; for compounds **2**, **3** and **5**: QAD > WAD > SAD > JSD; for compound **4**: WAD > SAD > JSD > QAD; for compounds **6** and **7**: SAD > WAD > JSD > QAD. In comparison with SAD, the contents of compounds **2**–**5** and **7** in WAD were higher than those in SAD, but the contents of ephedrine and formononetin decreased after SH and PR were added into SAD; the contents of compounds **1**–**3** in QAD were higher than those in SAD, and other compounds decreased after PR, SC, GF, CS were added into SAD; the contents of compounds **2**–**7** in JSD were lower than those in SAD, but the contents of ephedrine increased after AR was added into SAD. And the total content of the seven bio-active constituents in QAD was highest in the four TCM formulae, which supported the interpretation that QAD had the best therapeutic effect on asthmatic mice induced by ovalbumin sensitization and RSV infection [11,12].

**Table 4:** Contents of the seven studied compounds in the investigated samples (*n* = 3).

Sample No.	Content (mg/g)							Total
	1	2	3	4	5	6	7	
SAD	2.24	13.28	6.79	1.25	0.59	1.23	12.18	37.56
WAD	2.23	19.12	11.69	1.41	0.61	1.05	12.41	48.52
QAD	2.99	24.82	12.77	0.71	0.75	0.7	7.19	49.93
JSD	2.39	10.69	4.85	1.08	0.42	0.72	8.66	28.81

These results demonstrated that SH and PR could significantly promote the dissolution of amygdalin (**2**) in AS and liquiritin (**3**) in GR during their preparation procedure; PR, SC, GF and CS could greatly improve the dissolution of ephedrine (**1**) in EH, amygdalin (**2**) in AS and liquiritin (**3**) in GR, and obviously reduce the dissolution of benzoic acid (**4**) in AS, formononetin (**6**) and glycyrrhizic acid (**7**) in GR; AR might obviously reduce the dissolution of formononetin (**6**) and glycyrrhizic acid (**7**) in GR. The results also suggested that some drug-drug interactions exist, such as depression and enhancement of extractive rate of the bioactive components in SAD and its series of formulae during their preparation procedure. Thus, when SAD and its series of formulae are used for disease treatment, the relative proportions of the herbs should be fixed to avoid any changes of bioactive component contents.

## 3. Experimental

### 3.1. Plant Materials

Ephedrae Herba (the dried herbaceous stem and root of *Ephedra sinica* Stapf) was collected from Xinjiang Province; Armeniacae Semen Amarum (the dried ripe seed with coat of *Prunus armeniaca* L. var. *ansu* Maxim.) and Gypsum Fibrosum (GaSO_4_·2H_2_O) were collected from Shandong Province; Glycyrrhizae Radix et Rhizoma (the dried root and rhizome of *Glycyrrhiza uralensis* Fisch.) was collected from Ningxia Province; Schizonepetae Herba (*Schizonepeta tenuifolia* Briq.) and Platycodonis Radix (*Platycodon grandiflorum* (Jacq.) A. DC.) were collected from Anhui Province; Pinelliae Rhizoma (*Pinellia ternate* (Thunb.) Breit.) was collected from Sichuan Province; Camelliae sinensis Folium (*Camellia sinensis* (L.) O. Kuntze) was collected from Jiangsu Province; Schisandrae Chinensis Fructus (*Schisandra chinensis* (Turcz.) Baill.) and Asari Radix et Rhizoma (*Asarum heterotropoides* Fr. Schmidt var. *mandshuricum* (Maxim.) Kitag) were collected from Liaoning Province. The crude herbs were identified by Professor Dekang Wu (Department of Pharmacognosy, Nanjing University of Chinese Medicine), and voucher specimens were deposited in the Herbarium of Nanjing University of Chinese Medicine, China.

### 3.2. Chemicals and Reagents

The reference standards of ephedrine, amygdalin, liquiritin and formononetin were obtained from National Institute for the Control of Pharmaceutical and Biological Products of China (Beijing, China). Isoliquiritin and glycyrrhizic acid were purchased from Shanghai Winherb Medical Science Co., Limited (Shanghai, China). Benzoic acid was provided by our laboratory and its structure was determined by ^1^H-NMR, ^13^C-NMR, MS and UV spectra (purity > 98%). The chemical structures of these reference compounds are shown in Figure 1. Acetonitrile (HPLC grade) and methanol (HPLC grade) were obtained from Tedia Inc. (Fairfield, OH, USA). EtOAc and ethanol were obtained from Hanbang Company (Huaian, China). Unless specified elsewhere, all reagents were used without further purification. Distilled and deionized water was purified by the superpure water system (Eped Technology Development Co., Ltd., Nanjing, China).

### 3.3. Preparation of Standard Solutions

A mixed standard stock solution containing ephedrine (**1**), amygdalin (**2**), liquiritin (**3**), benzoic acid (**4**), isoliquiritin (**5**), formononetin (**6**) and glycyrrhizic acid (**7**) was prepared in methanol. Working standard solutions were prepared by diluting the mixed standard stock solution with methanol to give seven different quantities within the ranges: 0.32–20.56 μg; 0.74–47.60 μg; 0.16–10.40 μg; 0.02–1.12 μg; 0.10–6.16 μg; 0.04–2.56 μg and 0.43–27.44 μg for calibration curves. The standard solutions were filtered through a 0.45 μm membrane prior to injection. All solutions were stored in a refrigerator at 4 °C before analysis.

### 3.4. Sample Preparation

*The preparation of SAD*: 4 g of EH, 10 g of AS and 4 g of GR were weighed in proportion and decocted together with boiling water twice for 2 h each time. The water extract solution was combined and the solvent was removed under vacuum below 65 °C till a certain volume at the ratio of 1:1 (w/w, weight of all herbs and the extracted filtrates) was reached, and then ethanol was added slowly with churning all the time until the ethanol content reached 80%. After standing for 24 h, the solution was filtered to remove the deposits and concentrated to a certain concentration under vacuum below 65 °C, and then partitioned with EtOAc. The EtOAc fraction was accurately weighed and dissolved in 10 mL of 80% methanol; sonicated twice for 30 min each time with a short interval; the extracted solution was diluted five times with methanol; afterwards, it was filtered through a 0.45 μm filter membrane. The filtrate was used as the sample solution of SAD. *The preparation of WAD*: 4 g of EH, 10 g of AS, 4 g of GR, 8 g of SH and 8 g of PR were weighed and prepared in the same way as SAD. *The preparation of QAD*: 4 g of EH, 10 g of AS, 4 g of GR, 10 g of PR, 6 g of SC, 20 g of GF and 10 g of CS were weighed and prepared in the same way as SAD. *The preparation of JSD*: 4 g of EH, 10 g of AS, 4 g of GR, and 3 g of AR were weighed and prepared in the same way as SAD.

### 3.5. Apparatus and Chromatographic Conditions

Analysis was performed on a Waters 2695 HPLC system, equipped with a binary pump, an autosampler and a column oven coupled to a variable wavelength DAD (Waters Corp., Milford, MA, USA). HPLC separation of terpenoids was achieved using a XTerra C_18_ column (4.6 × 250 mm, 5.0 μm, Waters). The separation of seven compounds was achieved by a linear gradient elution of a mobile phase containing acetonitrile (A) and a buffer solution (0.02 mol/L potassium dihydrogen phosphate and adjusted pH 3 using phosphoric acid) (B). The gradient profile was as follows: 0–15 min: linear 5%–8% of A (v/v); 15–30 min: isocratic 8% of A; 30–44 min: 8%–32% A; 44–50 min: 32%–95% of A. A re-equilibration period of 10 min was used between individual runs. Detection wavelength was set at 210 nm for reference compounds 1–2; 254 nm for 3, 4, 6, 7 and 360 nm for 5. Chromatography was carried out at 30 °C with a flow rate of 1.0 mL/min. An injection volume of 10 μL was used.

### 3.6. Validation of the Methods

The dilute solution of the reference compounds was further diluted to a series of concentrations with methanol to assess the limits of detection (LOD) and quantification (LOQ). The LOD and LOQ were determined at signal-to-noise (S/N) ratios of 3 and 10, respectively. The intraday and interday precision was determined by analyzing calibration samples during a single day and on three consecutive days, respectively. To confirm the repeatability, six different working solutions were analyzed. The R.S.D. was taken as a measure of precision and reproducibility. A recovery test was used to evaluate the accuracy of the method. In the test, reference compounds were added to SAD at low (80% of the known amounts), medium (same as the known amounts) and high (120% of the known amounts) levels.

## 4. Conclusions

In the present study, chromatograms for determination of the seven major bio-active components in SAD and its series of formulae were developed and applied by using an HPLC-DAD method. Method validation of linearity, precision, repeatability, stability and accuracy were acceptable. The results demonstrated that the proposed method was able to detect comprehensive content changes after the relative herbs were combined into San-ao decoction or its series of formulae. The research also provided some experimental reference data for clinic applications of these formulae.
